# Reliable and Fast Localization in Ambiguous Environments Using Ambiguity Grid Map

**DOI:** 10.3390/s19153331

**Published:** 2019-07-29

**Authors:** Gen Li, Jie Meng, Yuanlong Xie, Xiaolong Zhang, Yu Huang, Liquan Jiang, Chao Liu

**Affiliations:** School of Mechanical Science and Engineering, Huazhong University of Science and Technology, Wuhan 430074, China

**Keywords:** navigation, perceptual aliasing, ambiguous environment, Monte Carlo localization, Dynamic Bayes network

## Abstract

In real-world robotic navigation, some ambiguous environments contain symmetrical or featureless areas that may cause the perceptual aliasing of external sensors. As a result of that, the uncorrected localization errors will accumulate during the localization process, which imposes difficulties to locate a robot in such a situation. Using the ambiguity grid map (AGM), we address this problem by proposing a novel probabilistic localization method, referred to as AGM-based adaptive Monte Carlo localization. AGM has the capacity of evaluating the environmental ambiguity with average ambiguity error and estimating the possible localization error at a given pose. Benefiting from the constructed AGM, our localization method is derived from an improved Dynamic Bayes network to reason about the robot’s pose as well as the accumulated localization error. Moreover, a portal motion model is presented to achieve more reliable pose prediction without time-consuming implementation, and thus the accumulated localization error can be corrected immediately when the robot moving through an ambiguous area. Simulation and real-world experiments demonstrate that the proposed method improves localization reliability while maintains efficiency in ambiguous environments.

## 1. Introduction

**Figure 1:**
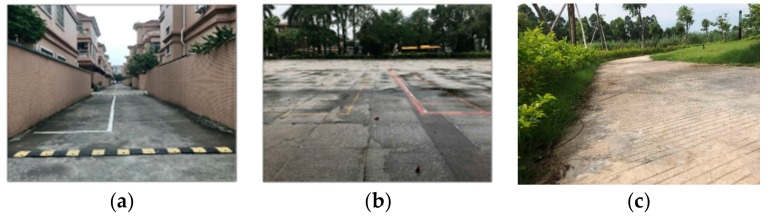
Ambiguous environments: (**a**) long corridor; (**b**) empty space; (**c**) tangled thicket.

For an autonomous robot, reliable and fast localization is a critical prerequisite during navigation. However, ambiguous environments may cause perception aliasing of the external sensors [1,2], which makes it a challenging task for a robot to accurately determine its pose or even leads to localization failure. As demonstrated in Figure 1*,* the ambiguous environments are specified as the environments that contain ambiguous areas, including long corridors, empty space, and tangled thicket. In such environments, however, there still exist some unambiguous areas that can help the robot to locate itself. For this end, constructing a localization method on the basis of the modeled ambiguity of the environment can increase the localization reliability without loss of efficiency.

For localization procedure of a robot, global localization is firstly performed to obtain the initial pose by using the observation data from external sensors, followed by the pose tracking which enables the robot to rapidly track its pose [3]. To be more specific, pose tracking consists of two steps, i.e., prediction and update. In the prediction step, based on the odometry motion model, the pose increment obtained by internal sensors is incorporated into the results of the previous localization moment to predict the current pose. As for the update, the localization error of the predicted pose is corrected by the observation data of external sensors, which respects the observation model to produce the final localization results of the current moment.

Recently, most of the existing localization methods [4,5,6,7,8,9] only rely on the external sensors in the update step, and it remains a tough job to satisfy the practical requirements when rectifying the localization error in an ambiguous environment. This is because the external sensors may suffer from perceptual aliasing, which implies that the observation data captured at different pose are difficult to distinguish due to the environmental ambiguity. Consequently, the localization error of the prediction step may accumulate as the robot moves through an ambiguous area of the environment. Even if the robot reaches an unambiguous area, the accumulated localization error is often too large to be corrected by those methods.

To solve the localization problem in an ambiguous environment, additional facilities such as artificial reflectors [10,11] and wireless sensor networks [12] have attracted increasing attention. With additional facilities, these methods require additional maintenance costs and need to modify the existing environment, which are not unreasonable for some practical usage. Assuming that a robot will leave the area where perceptual aliasing occurs and enters an unambiguous area, the pose of the robot can be recovered by a global localization method. In an unambiguous area, although global localization method can be used to estimate the pose of a robot according to the observation data captured by external sensors, the operating time of global localization is much longer than that of pose tracking. In addition, it is necessary to solve the detection problem of when the robot enters the unambiguous area. Additional information [13,14] except for the readings of internal sensors is utilized in the prediction step of pose tracking to allow more reliable pose prediction. However, their methods are impractical to solve the robot localization problem in ambiguous environments due to the fact that they do not explicitly consider the ambiguity of an environment and such environment property contains useful information for robot localization.

For robot navigation, the adaptive Monte Carlo localization (AMCL) method is able to achieve effective and fast robot localization in different environments [6,7,8,9] and the particle representation used in AMCL has included some effects of ambiguity. However, due to the limitation of particle number, the inaccurate motion model and observation model, it cannot cope with long term perception aliasing. In this paper, the AGM which explicitly models the ambiguity of an environment is utilized to improve the standard AMCL so that it can correct the accumulated localization error after robot moving through an ambiguous area and ultimately achieve a fast and reliable robot localization. The main contributions of this paper are summarized as follows:

- The AGM is achieved by proposing a new localizability evaluation method called average ambiguity error (AAE) so that the possible localization error at a given pose can be estimated, and those unambiguous areas of an environment can be identified.
- By integrating the AGM, the standard Dynamic Bayes network (DBN) for robot localization is improved to model the localization problem in ambiguous environments. Moreover, a new motion model referred to as portal motion model is implemented to obtain more reliable pose prediction in ambiguous areas.
- Based on the improved DBN, the AGM-based adaptive Monte Carlo localization (AGM-AMCL) method is derived to achieve fast probability inference.
- Simulation and real-world experiments validate the effectiveness of the AGM and the AGM-AMCL method, which can reliably locate a robot with guaranteed efficiency in three different ambiguous environments.

The remainder of this paper is constructed as follows: Section 2 presents a range of related work in terms of localizability evaluation and reliable localization. Section 3 provides the AGM and its detailed implementation. In Section 4, we describe the improved DBN, and then we derive the portal motion model and the AGM-AMCL. Finally, in Section 5, simulations and real-world experiments are conducted to evaluate the performance of the AGM and the AGM-AMCL method. Finally, some conclusions are offered in Section 6.

## 2. Related Work

Considering the key issue we attempt to solve in this paper, we will focus on the related work of localizability evaluation and reliable localization in ambiguous environments. To the best of our knowledge, there are few articles explicitly dealing with the localization problem in ambiguous environments. Global localization methods and optimized pose tracking methods can be used to relocate a robot after the robot leaving an ambiguous area and entering an unambiguous area. Therefore those methods were introduced in the reliable localization part.

### 2.1. Localizability Evaluation

Evaluating the localizability in an environment is to quantitatively estimate the achievable localization accuracy of a given pose. In general, localizability evaluation methods are not related to the property of an environment, therefore they are applicable in both ambiguous environments and unambiguous environments. Fisher information matrix (FIM) is a useful indicator for evaluating environmental localizability. Based on the Cramér-Rao lower bound, Censi [15,16] used FIM to represent the theoretical accuracy limit of a localization method employing range-finder data and attempted to use FIM to analyze the achievable accuracy of pose tracking. FIM has no analytical solution if an environment is represented by the occupancy grid map. To solve this problem, a discrete FIM [17] was proposed, which was obtained by simulated scan rays from an occupancy grid map. Afterwards, Irani et al. [18] presented a path planning method for a mobile manipulator based on discrete FIM. The localizability evaluation methods based on FIM can be used to evaluate the theoretical localization accuracy limit of a certain position, however, it is difficult for FIM to estimate the localization accuracy in a real localization process. This is because, under a given environment, the FIM is only related to the robot’s pose, without considering the environmental similarity of different poses and the observation model used in the actual localization process.

In addition to the localizability evaluation based on the FIM, there have been some related studies in recent years. In [19], a novel strategy for incremental sampling-based motion planning was proposed, which introduced a new evaluation of localizability using the trace change rate of covariance matrix from the prediction step to the update the step of an extended Kalman filter. Zhen et al. [20] proposed a method to evaluate the localizability based on the constraints provided by a sensor measurement. The above methods can evaluate the environmental localizability relatively while the evaluation value itself is meaningless for estimating localization error. In [21], to evaluate the localizability, a vehicle was driven through the designated environment and an error model was learned. However, their method takes a long time to experiment and is not suitable for large scale environments. Javanmardi et al. [22] adopted four types of criteria to evaluate the capability of maps for localization. Based on their criteria, the localizability of a map can be comprehensively evaluated. Our method differs from theirs in the sense that the observation model adopted in the real localization process and the sensor measurement uncertainties are involved in our evaluate method.

### 2.2. Reliable Localization

In recent years, many localization methods have been proposed to enhance localization reliability. One of the commonly used methods is adding additional facilities. Our previous work [10] proposed a new observation model for MCL, which improves the robustness of localization by reliable reflector prediction in the ambiguous environments. Beinhofer et al. [23] addressed the data association problem by deploying a limited number of uniquely identifiable artificial landmarks. A practical approach to landmark placement was presented in [24] that minimizes the localization errors. Besides, wireless sensor networks [12], radio frequency identification [25,26], etc. were also applied to achieve reliable localization. However, the additional facilities are costly for placement and maintenance, which limits the abovementioned method for potential applications.

Once localization fails, re-localization can be achieved through global localization, thereby improving the reliability of localization. A particle filter improved by a particle swarm optimization algorithm was proposed in [27]. With their method, more robust global localization can be achieved using fewer particles. A laser scan matching with an improved probabilistically-motivated Maximum Likelihood Estimation algorithm is adopted to obtain the global optimum position [2], which can reach an updating rate of 2 Hz in a feature-poor environment. A novel branch-and-bound multiresolution global localization method using a multiresolution Gaussian mixture map was proposed in [28] which was used for real-time localization in urban environments. By finding correspondences between a set of sparse features detected in two occupancy grid maps, a map to map matching method was designed [29], which can be used for global localization. In [30], a differential evolution approach was tested for global localization in an environment with different types of occlusions, which used three different cost functions based on a non-symmetric fitness function. The above methods attempt to achieve reliable and fast global localization. However, due to the need for global optimization, their running time is still longer than pose tracking. In [31] and [32], classifiers for pose recognition were trained by the machine learning method. Through such classifiers, the global pose of a robot can be identified quickly. However, those methods rely on reliable feature extraction methods, and wrong feature extraction may result in large localization errors.

By optimizing the prediction step of pose tracking, it can generate more accurate pose samples, also called particles, and improve the reliability of localization. Gutmann et al. [33] used sensor resetting method to generate some pose particles in the prediction step. The number of particles relies on the ratio between the short term and the long term average observation likelihood. Building on the basic Monte Carlo localization algorithm, a robust localization algorithm called Mixture-MCL was developed [34], which attempted to generate pose samples according to the observation model using a set of learned kd-trees in the prediction step to complement the odometry motion model. However, this method takes a large amount of memory, and as the size of the environment grows, the amount of memory required grows rapidly. Zhang et al. [13] proposed the concept of similar energy region. Extra pose samples can be generated in similar energy region to obtain a reliable pose prediction. Oh et al. [14] developed an Augmented Motion model, which used the semantic information available in maps to bias the motion model toward areas of higher probability. In [35], by using a global hypothetical pose set to represent an environment, a novel qualitative motion model is proposed to generate extra pose hypothesis. In [13,14,33,34,35], additional information is used in the prediction step to generate extra pose samples which may closer to the robot true pose. Once the accumulated localization error is too large, extra pose samples may compensate for such error and improve the reliability of localization. However, their methods do not explicitly consider the ambiguous nature of an environment, it is very difficult for these methods to achieve reliable localization in ambiguous environments. Inspired by the abovementioned methods, additional information from the AGM is exploited to improve the prediction step of the AMCL method, which allows our method to generate some extra particles at a proper time with proper distribution and effectively improves the reliability of localization.

## 3. AGM

AGM is used for modeling the ambiguity of an environment, such that we can figure out which positions suffer from perceptual aliasing and how much localization error will be caused by perceptual aliasing at those positions. Analogical to the occupancy grid map, AGM has multiple cells, each of which measures the localization error resulting from perceptual aliasing. In this paper, we refer the aforesaid localization error to the AAE, and it is crucial to build the AGM. Practically, it is noticed that a robot cannot distinguish two different poses effectively due to the similar surroundings of these poses or the considerable sensor noise or poor observation model [36]. Considering that, the formulation of AAE should be related to the environment as well as the external sensor and the constructed observation model.

Define x=(p,θ) as a pose with p=(x,y) used to denote a position in two-dimension. Let $z_{x}$ be the observation data obtained by the external sensor of a robot at the pose x. Using the observation data $z_{x}$ and an input pose y, the likelihood function of an observation model is denoted by:

(1) $$L_{x}(y)≜P(z_{x}|y)$$

From Equation (1), we conclude that $L_{x}(x)$ represents the likelihood value when the input pose y is equal to the robot’s true pose. For a general pose y if the maximum value of $L_{x}(y)$ is identical with $L_{x}(x)$, the robot’s pose can be accurately obtained through maximum likelihood estimation. However, in an ambiguous environment, there are other values of y that makes $L_{x}(y)$ equal or bigger than $L_{x}(x)$. In this situation, it is difficult to distinguish pose x and pose y based on this likelihood function. Thus, in this paper, the probability of a robot’s true pose x confused with some other pose y can be defined as:

(2) $$P_{y|x}^{A}≜P(L_{x}(y)+\varepsilon \geq L_{x}(x))$$

where ε is a small threshold to compensate the unmodeled factors of the likelihood computation.

Given the observation data $z_{x}$, the likelihood value of $L_{x}(y)$ can be uniquely determined. Thus probability $P_{y|x}^{A}$ is rely on the distribution of $z_{x}$ and Equation (2) can be expressed as:

(3) $$P_{y|x}^{A}=\;\int_{z_{x}}1_{\left(L_{x}(y)+\varepsilon \geq L_{x}(x)|z_{x}\right)}(z_{x})P(z_{x})dz_{x}$$

where $P(z_{x})$ is the distribution of observation data at the pose x and $1_{\left(L_{x}(y)+\varepsilon \geq L_{x}(x)|z_{x}\right)}(z_{x})$ denote an indicator function, i.e.:

(4) $$1_{\left(L_{x}(y)+\varepsilon \geq L_{x}(x)|z_{x}\right)}(z_{x})=\left\{\begin{array}{l} 1\;L_{x}(y)+\varepsilon \geq L_{x}(x)|z_{x}\;\\ 0\;L_{x}(y)+\varepsilon <L_{x}(x)|z_{x} \end{array}\right.$$

Since $P_{y|x}^{A}$ cannot be determined analytically, we employ Monte Carlo integral to achieve its approximation as:

(5) $$P_{y|x}^{A}\approx \;\frac{1}{N}\sum_{i=1}^{N}1_{\left(L_{x}(y)+\varepsilon \geq L_{x}(x)|z_{{}_{x}}^{i}\right)}(z_{{}_{x}}^{i})$$

where $z_{x}^{i}$ denotes the *i*-th sample of the observation data, N denotes the total number of samples.

Now we define the AAE of a given pose x as:

(6) $$aae(x)=\frac{\sum_{\Delta_{i}\in M}d(x+\Delta_{i},x)P_{x+\Delta_{i}|x}^{A}}{\sum_{\Delta_{i}\in M}P_{x+\Delta_{i}|x}^{A}}$$

where M is a set of the incremental pose, $\Delta_{i}≜(\Delta x_{i},\Delta y_{i},\Delta \theta_{i})$ denotes the *i*-th element in M, $d(x+\Delta_{i},x)$ denotes the difference between $x+\Delta_{i}$ and x, which is expressed by:

(7) $$d(x+\Delta_{i},x)=\sqrt{\Delta x_{i}^{2}+\Delta y_{i}^{2}}+\xi |\Delta \theta_{i}|$$

where ξ is a coefficient factor used to convert the orientation difference into the distance difference.

From Equation (6), it is known that the AAE of a pose represents the weighted average localization error caused by the ambiguity between the true pose and the other poses which are contained by M. Large AAE value of a given pose means that a large localization error may be induced after a robot moving through that pose. Moreover, by exploiting the AAE value of a trajectory, the accumulated localization can be estimated in some way and this property is used in our localization method in the following section.

Then, based on Equation (6), the AAE of a position can be derived directly:

(8) $$aae(p_{x})=\frac{1}{n_{\theta }}\sum_{\theta =0{}^{\circ}}^{360{}^{\circ}}aae(x(p,\theta ))$$

where $n_{\theta }$ denotes the number of discrete θ.

**Figure 2:**
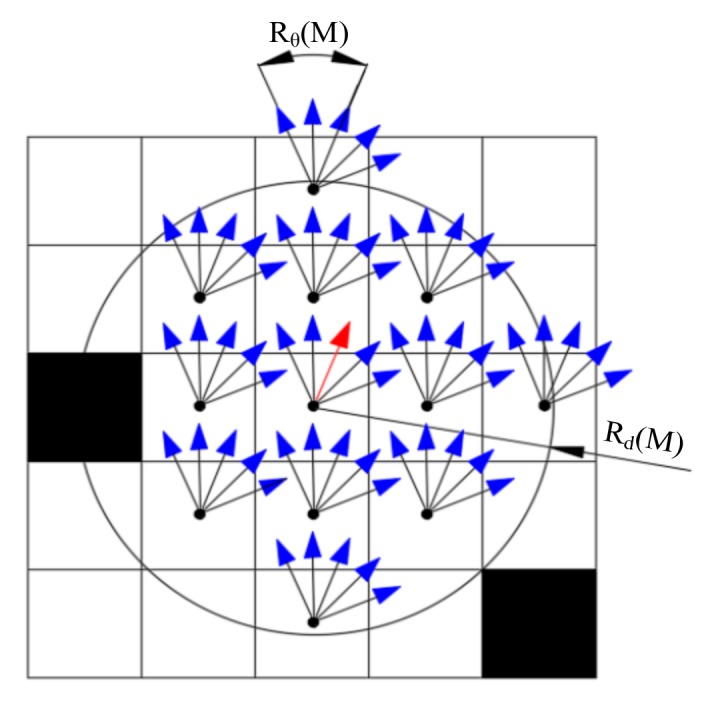
Schematic diagram of M. The black grids and white grids indicate the occupied cells and free space cells, separately. The $R_{d}(M)$ and $R_{\theta }(M)$ are marked on this figure. The position and the direction of the red arrow represent the pose x to be evaluated. Those blue arrows represent those poses derived from the incremental pose set M and x according to $x+\Delta_{i}$.

Figure 2 shows a schematic diagram of M. Defining $R_{d}(M)$ and $R_{\theta }(M)$ as the distance range and the orientations range of the set M, respectively, we have:

(9) $$M=\{\Delta_{i}≜(\Delta x_{i},\Delta y_{i},\Delta \theta_{i})|\sqrt{\Delta x_{i}^{2}+\Delta y_{i}^{2}}\leq R_{d}(M)\wedge \left|\Delta \theta_{i}\right|\leq R_{\theta }(M)\}$$

In this paper, in accordance with occupancy grid map, $\Delta x_{i}$ and $\Delta y_{i}$ are pre-selected as an integer multiple of the grid map resolution and $\Delta \theta_{i}$ is selected as an integer multiple of 3° to make the trade-off between the efficiency and accuracy.

To derive a solvable solution for AAE determined by Equation (8), we need to focus on the sampling method of $P(z_{x})$ and the configuration of ξ, $R_{d}(M)$ and $R_{\theta }(M)$. In addition, considering that a 2-D laser range finder is used as the external sensor in this paper, the following method for computing $P(z_{x})$ is based on the 2-D laser range finder. First, sampling with a large amount of data is time-consuming and unrealistic in a large-scale environment. As studied in [37], the 2-D laser range finder and the beam model are suggested to be used as the external sensor and observation model for the practical implementation, respectively. Then we can adopt the following feasible and effective method to obtain observation data samples: (1) build the occupancy grid map of an environment using SLAM method; (2) determine the parameters of the beam model by real observation data using learning method presented in [37]; (3) obtain the observation data samples at various poses through simulation. Second, the ξ is expressed by $\xi =\chi_{1}/rad(\chi_{2})$, where $\chi_{1}$ and $\chi_{2}$ denotes the maximum tolerable distance error and the maximum tolerable orientation error for reliable localization, separately. Finally, by using the inequality $aae(x_{p})\leq R_{d}(M)+\xi R_{\theta }(M)$, which can be derived from Equations (6) and (7), we can obtain the range constraints for M, i.e., $\chi_{1}\leq R_{d}(M),\;\chi_{2}\leq R_{\theta }(M)$. More accuracy localizability evaluation can be obtained through a larger range of M while it is time-consuming. Therefore, we select $R_{d}(M)=\chi_{1}$ and $R_{\theta }(M)=\chi_{2}$.

## 4. AGM-AMCL

**Figure 3:**
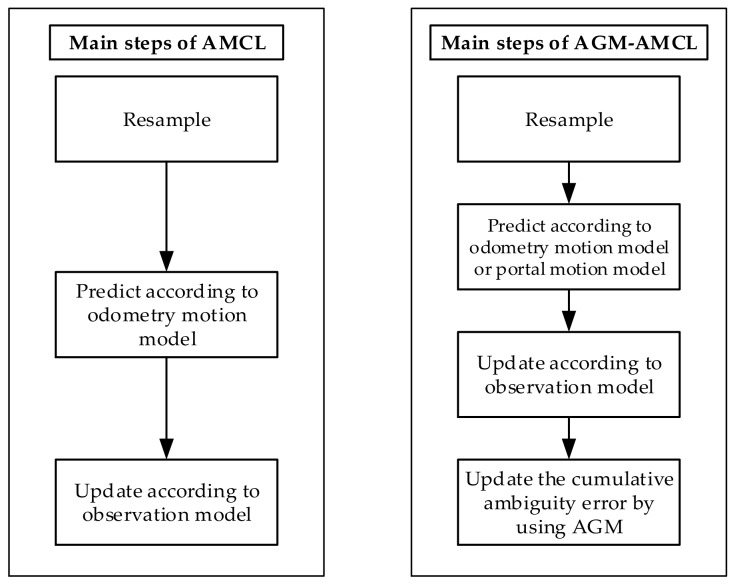
Main steps of AMCL and our method.

As shown in Figure 3, the standard AMCL [38] mainly contains three steps: resampling step, prediction step, and update step. In the resampling step, particles are sampled according to the likelihood of each particle. Then new particles are generated in the prediction step based on the odometry motion model. After that, the likelihood of each particle is evaluated by the observation model in the update step. In addition to those three steps, our method also includes a step for estimating the accumulated ambiguity error using the AGM. Moreover, in the prediction step of our method, the portal motion model is used to generate extra particles in an adjacent unambiguous area if a robot is in an ambiguous area. Due to the extra particles, large localization error can be corrected as soon as the robot enters the adjacent unambiguous areas.

In fact, our method can be derived from a DBN which models the robot localization problem in unambiguous environments. In the following part, the derivation of our method, as well as the portal motion model and the estimation method for the accumulated localization error using the AGM, will be detailed introduced.

### 4.1. Standard DBN for Localization

Given a prior occupancy grid map $m_{g}$, the robot localization can be modeled as a probabilistic inference problem:

(10) $$Bel(x_{t})=P(x_{t}|z_{1:t},u_{1:t},m_{g})$$

where $x_{t}$ denotes the robot pose at a time t, $z_{1:t}$ is the sequence of observations, $u_{1:t}$ denotes a sequence of motion control commands or odometry measurements.

**Figure 4:**
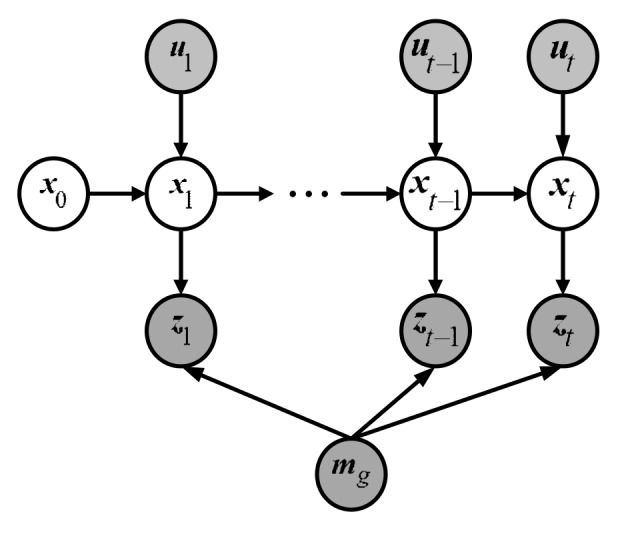
Standard DBN for localization problem.

Figure 4 demonstrates the standard DBN [39] for robot localization which contains two importance independent assumptions: Markov independence of the odometry and Markov independence of the observation.

According to the independent assumptions embedded in the standard DBN, we can simplify the robot pose belief that presented in Equation (10) as:

(11) $$Bel(x_{t})=\eta P(z_{t}|x_{t},m_{g})\int_{x_{t-1}}P(x_{t}|x_{t-1},u_{t})Bel(x_{t-1})dx_{t-1}$$

where η is a normalization coefficient, $P(x_{t}|x_{t-1},u_{t})$ and $P(z_{t}|x_{t},m_{g})$ are corresponding to the motion model and observation model, respectively. To fast infer the robot’s pose belief in Equation (11), AMCL [38] can be used with the KLD-sampling method regulating the particle set size.

Because of perception aliasing, the localization error accumulates rapidly when a robot moves through an ambiguous area. However, the DBN, shown in Figure 4, does not explicitly consider the accumulated localization error caused by perception aliasing so that it is not suitable for modeling the robot localization problem in ambiguous environments. For this reason, an improved DBN is proposed.

### 4.2. Improved DBN

**Figure 5:**
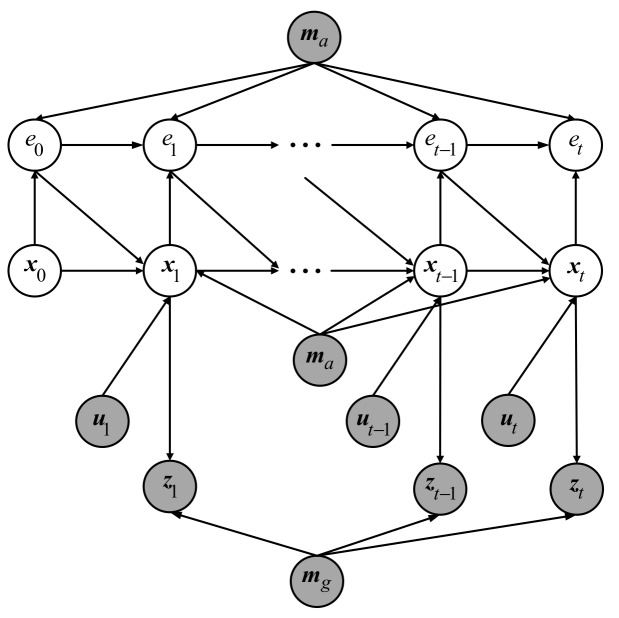
Improved DBN for localization problem. The AGM node ($m_{g}$) and the cumulative ambiguity error node ($e_{t}$) are introduced.

The improved DBN is shown in Figure 5, where $m_{g}$ stands for the occupancy grid map and $m_{a}$ is the corresponding AGM, $e_{t}$ is the cumulative ambiguity error at time *t*, which reflects the accumulated localization error caused by perception aliasing.

To infer the robot’s pose, as well as the accumulated localization error, the robot localization in ambiguous environments is expressed as:

(12) $$Bel(x_{t},e_{t})=P(x_{t},e_{t}|z_{1:t},u_{1:t},m_{a},m_{g})$$

Also, based on the independent assumptions embedded in the improved DBN, we have:

(13) $$\begin{array}{ll} Bel(x_{t},e_{t}) & =P(x_{t},e_{t}|z_{1:t},u_{1:t},m_{a},m_{g})\\ & =\eta P(z_{t}|x_{t},m_{g})P(x_{t},e_{t}|z_{1:t-1},u_{1:t},m_{a},m_{g})\\ & =\eta P(z_{t}|x_{t},m_{g})\iint P(x_{t},e_{t},x_{t-1},e_{t-1}|z_{1:t-1},u_{1:t},m_{a},m_{g})dx_{t-1}de_{t-1}\\ & =\eta P(z_{t}|x_{t},m_{g})\;\iint P(x_{t},e_{t}|x_{t-1},e_{t-1},u_{t},m_{a})P(x_{t-1},e_{t-1}|z_{1:t-1},u_{1:t-1},m_{a},m_{g})dx_{t-1}de_{t-1}\;\\ & =\eta P(z_{t}|x_{t},m_{g})\iint P(e_{t}|x_{t},e_{t-1},m_{a})\;P(x_{t}|x_{t-1},e_{t-1},u_{t},m_{a})Bel(x_{t-1},e_{t-1}) \end{array}$$

As compared to Equation (11), we conclude that Equations (11) and (13) share the same observation model $P(z_{t}|x_{t},m_{g})$. A different motion model $P(x_{t}|x_{t-1},e_{t-1},u_{t},m_{a})$ considering the cumulative ambiguity error $e_{t-1}$ and the $m_{a}$ are imposed in the improved DBN. Meanwhile, Equation (13) has an additional term $P(e_{t}|x_{t},e_{t-1},m_{a})$ to estimate the cumulative ambiguity error. It is noted that the multiple integrations in Equation (13) cannot be derived analytically. In our paper, the AMCL is adopted to inference the robot’s pose as well as the accumulated localization error, which utilized a set of weighted particles to estimate the probability distribution. Therefore, the probability distribution $P(x_{t},e_{t},x_{t-1},e_{t-1}|z_{1:t-1},u_{1:t},m_{a},m_{g})$ can be reformulated according to [40] as:

(14) $$P(x_{t},e_{t},x_{t-1},e_{t-1}|z_{1:t-1},u_{1:t},m_{a},m_{g})=\sum_{i=1}^{n_{P}}\omega_{t}^{i}\delta ([x_{t},e_{t},x_{t-1},e_{t-1}]-[x_{t}^{i},e_{t}^{i},x_{t-1}^{i},e_{t-1}^{i}])$$

where $\left[x_{t}^{i},e_{t}^{i},x_{t-1}^{i},e_{t-1}^{i}\right]$ denotes a sample of robot state also called particle, $\omega_{t}^{i}$ stands for the *i*-th particle weight, δ is the Dirac delta function and $n_{p}$ is the number of particles.

Substituting Equation (14) into the second part of Equation (13) yields:

(15) $$\begin{array}{l} P(x_{t},e_{t}|z_{1:t},u_{1:t},m_{a},m_{g})=\iint P(x_{t},e_{t},x_{t-1},e_{t-1}|z_{1:t-1},u_{1:t},m_{a},m_{g})dx_{t-1}de_{t-1}\\ =\sum_{i=1}^{n_{P}}\omega_{t}^{i}\iint \delta ([x_{t},e_{t},x_{t-1},e_{t-1}]-[x_{t}^{i},e_{t}^{i},x_{t-1}^{i},e_{t-1}^{i}])dx_{t-1}de_{t-1}\\ =\sum_{i=1}^{n_{P}}\omega_{t}^{i}\delta ([x_{t},e_{t}]-[x_{t}^{i},e_{t}^{i}]) \end{array}$$

### 4.3. Portal Motion Model for Improved DBN

Intuitively, the prediction step of the AMCL generates some particles in an unambiguous area which is adjacent to the ambiguous area, the update and resampling steps of the AMCL method will make the particles immediately converge to the robot’s true pose when the robot leaves the ambiguous area. To accommodate that, we rewrite the motion model $P(x_{t}|x_{t-1},e_{t-1},u_{t},m_{a})$ as:

(16) $$P(x_{t}|x_{t-1},e_{t-1},u_{t},m_{a})=(1-\alpha_{t})P_{odo}(x_{t}|x_{t-1},u_{t})+\alpha_{t}P_{portal}(x_{t}|x_{t-1},e_{t-1},m_{a})$$

where $P_{odo}(x_{t}|x_{t-1},u_{t})$ is the odometry motion model, $\alpha_{t}$ is a weighting factor and $P_{portal}(x_{t}|x_{t-1},e_{t-1},m_{a})$ is the portal motion model to generate the particles in an adjacent unambiguous area, which is defined as:

(17) $$P_{portal}(x_{t}|x_{t-1},e_{t-1},m_{a})=\{\begin{array}{ll} \eta_{hit}e^{-\frac{1}{2}{\left(x_{t}-\mu_{hit}\right)}^{T}\Sigma_{hit}^{-1}(x_{t}-\mu_{hit})}, & for\;aae(p_{x_{t}})\leq t_{a}\\ 0,\; & for\;aae(p_{x_{t}})>t_{a} \end{array}$$

where $\eta_{hit}$ is a normalization coefficient, $aae(p_{x_{t}})$ denotes the AAE of the position $p_{x_{t}}$ for the pose $x_{t}$, $t_{a}$ is a threshold and $aae(x_{t})\geq t_{a}$ implies the robot is in an ambiguous area. $t_{a}$ is set to $\chi_{1}$ which denote the maximum tolerable distance error. It is concluded from Equation (17) that given $x_{t-1}$, $e_{t-1}$ and $m_{a}$, $x_{t}$ obeys a truncated Gaussian distribution. Specifically, in an ambiguous area, the probability of $x_{t}$ is zero whereas it obeys a Gaussian distribution with a mean $\mu_{hit}$ and a covariance matrix $\Sigma_{hit}$ in an unambiguous area.

The portal motion model describes a state transition that a robot may be immediately moved to someplace in the adjacent unambiguous area regardless of the odometer measurement. Despite the physically impossible, it can be used to obtain more reliable pose prediction in an ambiguous environment so that the accumulated localization error can be corrected immediately when the robot reaches the adjacent unambiguous area. As a complement to the odometer motion model, the portal motion model explicitly indicates the effect of cumulative localization error caused by perception aliasing on prediction robot pose.

**Figure 6:**
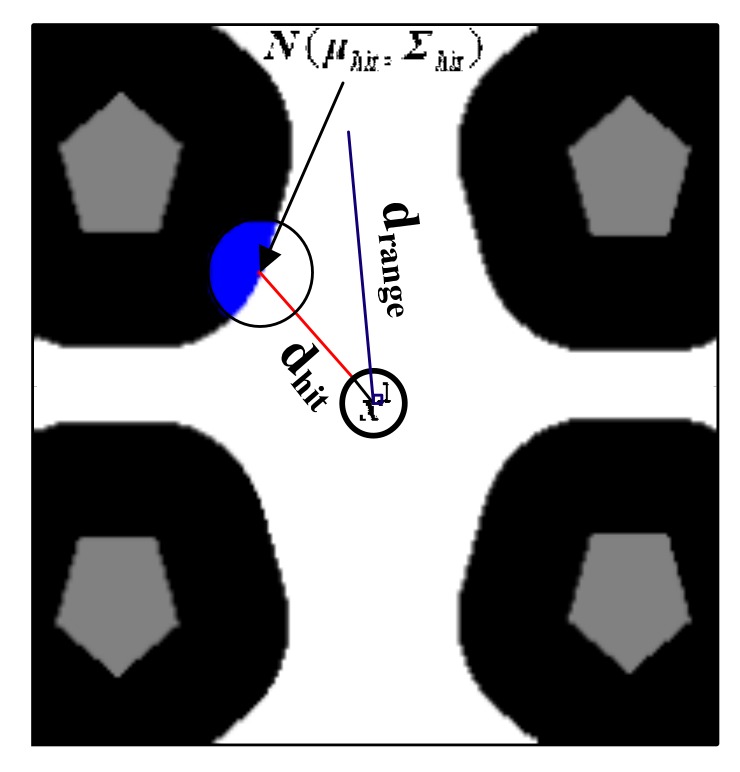
Schematic diagram of the portal motion model. The white area, black area, and gray area represent an ambiguous area, an unambiguous area, and area occupied by obstacles, respectively. $x^{1}$ is a pose sample. The blue transparent circle is the Gaussian distribution $N(\mu_{hit},\Sigma_{hit})$, and the blue opaque portion expresses the truncated Gaussian distribution of Equation (17).

A schematic diagram for the portal motion model is shown in Figure 6. We define $d_{range}≜e_{t-1}$ as the cumulative ambiguity error at the time t−1, and its length is shown with a blue line. Assuming the robot pose at the time t−1 is $x^{1}$ which is in the ambiguous area, $\mu_{hit}$ can be generated by a simulated beam with ray casting method [41]. Practically, the simulated beam continues unless the endpoint of the beam hits an unambiguous area or an obstacle or the length of the beam reaches $d_{range}$. As shown in Figure 6, the red line shows the simulated beam generated from $x^{1}$ and the length of the beam is represented as $d_{hit}$. if the beam ends in an unambiguous area, the position and the orientation of $\mu_{hit}$ can be determined by the position and orientation of the beam, respectively. And if the beam ends because of the other two conditions, the portal motion model is not enabled. Define $\sigma_{p}$ and $\sigma_{\theta }$ representing the adjustable standard deviation of the position and orientation, respectively. Large $\sigma_{p}$ and $\sigma_{\theta }$ means large uncertainties of the truncated Gaussian distribution. Then, we can calculate $\Sigma_{hit}$ by:

(18) $$\Sigma_{hit}=\left[\begin{array}{ccc} \sigma_{p}^{2} & 0 & 0\\ 0 & \sigma_{p}^{2} & 0\\ 0 & 0 & \sigma_{\theta }^{2} \end{array}\right]\left[\nu_{1},\nu_{2},\nu_{3}\right]\left[\begin{array}{ccc} 1 & 0 & 0\\ 0 & k_{hit} & 0\\ 0 & 0 & 1 \end{array}\right]{\left[\nu_{1},\nu_{2},\nu_{3}\right]}^{-1}$$

where $\nu_{1}$ is a unit vector with the same orientation as $x^{1}$, $\nu_{2}$ is the unit vector perpendicular to $\nu_{1}$, $\nu_{3}={\left(0,0,1\right)}^{T}$, $k_{hit}$ is a stretch factor that used to adjust the ratio between the feature vectors of the covariance matrix $\Sigma_{\mathrm{hit}}$ given by:

(19) $$k_{hit}=(\begin{array}{ll} k_{\max},\;for & \frac{d_{range}}{d_{hit}}\geq k_{\max}\\ \frac{d_{range}}{d_{hit}},\;for\; & else \end{array}$$

where $k_{\max}$ is the parameter to limit the ratio between the feature vectors of the covariance matrix $\Sigma_{hit}$.

**Figure 7:**
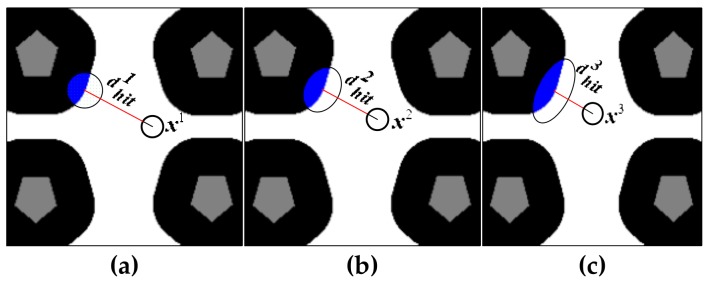
Changing process of $\Sigma_{hit}$. The uncertainty of $N(\mu_{hit},\Sigma_{hit})$ is increasing as a robot moves in an ambiguous area and more particles will be generated by the portal motion model.

Figure 7a–c depict the changing process of $\Sigma_{hit}$. It can be seen that as the pose of the robot changes from $x^{1}$ to $x^{3}$, $d_{hit}$ gradually decreases ($d_{hit}^{1}>d_{hit}^{2}>d_{hit}^{3}$). According to Equation (18), the uncertainties of the truncated Gaussian distribution in the direction perpendicular to the robot’s orientation gradually increase due to the decreasing of $d_{hit}$, as represented by the blue oval area. We adopt the KLD-sampling method such that few and relatively concentrated particles will be generated by the portal motion model when the robot is in an ambiguous area and is far away from an unambiguous area. Moreover, as the distance between the robot and the unambiguous area getting shorter, more dispersed particles will be generated by the portal motion model due to large uncertainties of $\Sigma_{hit}$. With an adaptive $\Sigma_{hit}$, we can reasonably add uncertainties for the portal motion model and improves the reliability of localization.

### 4.4. AGM-AMCL Implementation

For implementation, $P(e_{t}|x_{t},e_{t-1},m_{a})$ is designed using a deterministic function:

(20) $$e_{t}=\left\{ \begin{array}{lll} e_{t-1} & +\frac{aae(p_{x_{t}})}{\rho \cdot e_{t-1}+1} & for\;aae(p_{x_{t}})>t_{a}\\ 0\; & & for\;aae(p_{x_{t}})\leq t_{a} \end{array}\right.$$

where ρ≥0 and is a parameter to control the changing rate of $e_{t}$. By Equation (20), we show that $e_{t}$ in an ambiguous area and an unambiguous area is derived as $e_{t}=e_{t-1}+aae(p_{x_{t}})/\left(\rho \cdot e_{t-1}+1\right)$ and $e_{t}=0$, respectively.

Using the length of the beam $d_{hit}$, we schedule the $\alpha_{t}$ adaptively as:

(21) $$\alpha_{t}=(\begin{array}{lll} 0 & & for\;(d_{hit}=d_{range})\vee (d_{hit}=0)\\ 1 & -\frac{d_{hit}}{d_{range}} & for0<d_{hit}<d_{range} \end{array}$$

As demonstrated in Equation (21), if the beam ends due to the length restriction or the zero- length of $d_{hit}$ which means the beam end because of obstacle or the pose $x_{t}$ in an unambiguous area, $\alpha_{t}$ is assigned to zero, otherwise, it is regulated in real-time where $0<d_{hit}<d_{range}$ indicates that the robot is in an ambiguous area and an unambiguous area is found in the scope of $d_{range}$.

A detailed overview of the AGM-AMCL algorithm can be found in Algorithm 1. The output particle set is initialized as an empty set in Step 1. Step 2 declares a variable $C_{t}$ to store the ray casting result to avoid repetitive computation and is initialized from Step 3 to Step 5. Step 7 to Step 23 show the prediction step of our localization method which is derived from Equation (16). $\mu_{hit}$ and $d_{hit}$ are obtained by the ray casting method at step 10, $\alpha_{t}$ is calculated according to Equation (21) at step 15. At step 16, c is a binary variable sampled from a Bernoulli distribution with parameter $\alpha_{t}$. c=1 means that the prediction pose will be sampled from the portal motion model while c=0 means the prediction pose will be sampled from the odometry motion model. At step 18, $\Sigma_{hit}$ is computed according to Equation (18). Through step 19 and 20, the portal model is used to sample a prediction pose. According to Equation (20), the cumulative ambiguity error $e_{t}$ is updated at step 24. In order to improve the efficiency of the particles, the KLD sampling method presented in [38] is adopted at step 28.
**Algorithm 1 AGM-AMCL****Input:**$S_{t-1}=\left\{{\langle x_{t-1}^{\left[i\right]},e_{t-1}^{\left[i\right]},\omega_{t-1}^{\left[i\right]}\rangle }_{i=1}^{N_{t-1}}\right\},u_{t},z_{t},m_{g},m_{a}$**Output:**$\;S_{t}=\left\{{\langle x_{t}^{\left[i\right]},e_{t}^{\left[i\right]},\omega_{t}^{\left[i\right]}\rangle }_{i=1}^{N_{t}}\right\}$1:  $S_{t}\leftarrow \emptyset ,N_{x}\leftarrow 0,n\leftarrow 0,\beta \leftarrow 0$   // Initialization2:  $C_{t}=\left\{{\langle x_{hit}^{\left[i\right]},d_{hit}^{\left[i\right]},{\mathrm{tag}}^{\left[i\right]}\rangle }_{i=1}^{N_{t-1}}\right\}$   //For storing RAYCAST result3:  for $i\leftarrow 1,\ldots ,N_{t-1}$ do   //Initialize $C_{t}$
4:   ${\mathrm{tag}}^{\left[i\right]}\leftarrow 0$
5:  end for 6:  do7:   $j\leftarrow \;\mathrm{RESAMPLING}\;\left(S_{t-1}\right)$   //Resampling according to particle weight8:   $d_{\mathrm{range}}=e_{t-1}^{\left[j\right]}$
9:   if ${\mathrm{tag}}^{\left[j\right]}==0$ then10:    $\left(\mu_{hit},d_{hit}\right)\leftarrow \mathrm{RAYCAST}\left(x_{t-1}^{\left[j\right]},d_{\mathrm{range}},m_{a}\right)$
11:    $C_{t}[j]\leftarrow \left(x_{hit},d_{hit},1\right)$
12:   else13:    $\left(\mu_{hit},d_{hit}\right)\leftarrow C_{t}[j]$
14:   end if15:   $\alpha \leftarrow \;\mathrm{COMPUTEALPHA}\;\left(d_{hit},d_{\mathrm{range}}\right)$
16:   sample c according to Bernoulli_Distribution(
α) 17:   if  c==1
18:    $\Sigma_{hit}\leftarrow \;\mathrm{COMPUTESIGMAHITT}\;\left(d_{\mathrm{range}},d_{hit},x_{t-1}^{\left[j\right]}\right)$
19:    Create $P_{\mathrm{portal}}\left(x_{t}|x_{t-1}^{\left[j\right]},e_{t-1}^{\left[i\right]},m_{a}\right)$ according to $N\left(\mu_{hit},\Sigma_{hit}\right)$
20:    Sample    $x_{t}^{\left[n\right]}$ according to $P_{\mathrm{portal}}\left(x_{t}|x_{t-1}^{\left[j\right]},e_{t-1}^{\left[i\right]},m_{a}\right)$
21:   else22:    Sample $x_{t}^{\left[n\right]}$ according to $P_{odo}\left(x_{t}|x_{t-1}^{\left[j\right]},u_{t}\right)$
23:   end if24:   $e_{t}^{\left[n\right]}\leftarrow \;\mathrm{PREDICTAMBIGUITYERROR}\;\left(x_{t}^{\left[n\right]},e_{t-1}^{\left[j\right]},m_{a}\right)$
25:   $\omega_{t}^{\left[n\right]}\leftarrow P\left(z_{t}|x_{t}^{\left[n\right]},m_{a}\right)$ //Compute weight according to observation26:   $\beta \leftarrow \beta +\omega_{t}$
27:   $S_{t}\leftarrow S_{t}\cup \left\{\langle x_{t}^{\left[n\right]},e_{t}^{\left[n\right]},\omega_{t}\rangle \right\}$
28:   $N_{x}\leftarrow \mathrm{ADPTINGSAMPLESIZE}\left(x_{t}^{\left[n\right]}\right)$
29:   n←n+1
30:  while $n<N_{x}$ and $n<N_{\min}$
31:  for i←1,…,n do   //Normalize particle weight32:   $\omega_{t}^{\left[i\right]}\leftarrow \omega_{t}^{\left[i\right]}/\beta$
33:  end for34:  return $S_{t}$



## 5. Experiments and Results

In this section, we will verify the effectiveness of the AGM and the performance of the AGM-AMCL algorithm severally. Note that, if not particularly indicated, AAE represents the AAE of a position in this section.

### 5.1. Platform and Environments

**Figure 8:**
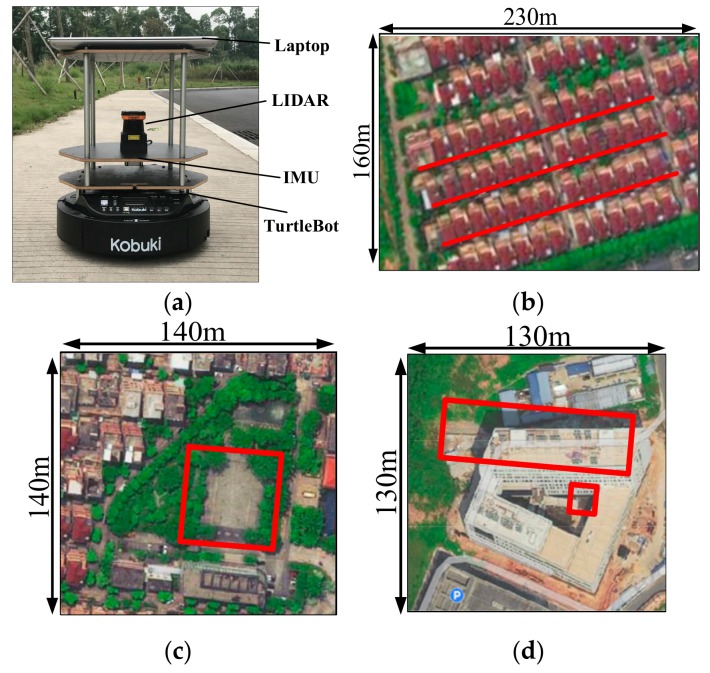
Experimental platform and real-world environments: (**a**) Turtlebot2 for data capturing; (**b**) scene A: long corridors; (**c**) scene B: empty space; (**d**) scene C: tangled thicket. The red lines in scene A show three parallel long corridors, and each corridor is surrounded by two parallel walls without prominent features. The red square in scene B shows an open square. The red square in scene C show area with tangled thicket.

The experimental platform and environments are shown in Figure 8, respectively. The used mobile robot named Turtlebot2 is equipped with a Hokuyo UTM-30LX LIDAR and an LPMS-RS232AL2 IMU, which serve as the external and internal sensor, separately. As depicted in Figure 8a,b, three different ambiguous environments with long corridors, open square and tangled thicket are considered for the real-world experiments.

### 5.2. AGM of Artificial Environments

**Figure 9:**
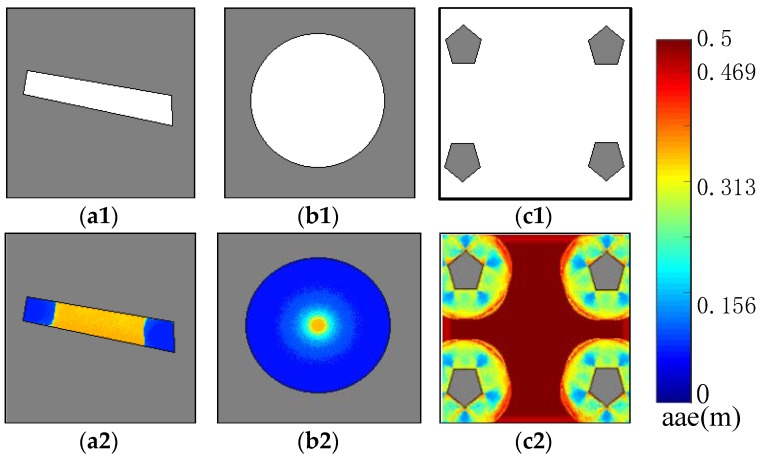
AGMs of virtual ambiguous environments: (**a1**–**c1**) are the occupancy grid maps of three artificial environments. (**a2**–**c2**) are the corresponding AGMs. AGMs show that the middle area of (**a1**), the center area of (**b1**) and the open area of (**c1**) get high AAE value because of their ambiguous.

To verify the effectiveness of the AGM, we draw several grid maps, in which the localizability of different position can be easily distinguished manually. In our research, the log-likelihood is used for observation likelihood computation. And is set to 0.5 which is a very small value for log-likelihood and is useful to compensate for some numerical calculation error. $\chi_{1}$ and $\chi_{2}$ were selected as 0.25 m and 5° respectively in this experiment, therefore the maximum AAE value is 0.5 m. As demonstrated in Figure 9, the top row and bottom row present three artificial occupancy grid maps and their corresponding AGM, respectively. Low AAE value (blue) of a cell means small localization error while high AAE value (red) means large localization error. When building these AGMs, the range error of the laser scan simulator was set to 0 m to simulate the laser scan data. The map shown in Figure 9(a1) is a long corridor. It can be seen from its AGM that the AAE value in both ends of the corridor is small, and the ε AAE value in the middle of the corridor is high, which is according with human experience. The map shown in Figure 9(b1), is a circle. When a robot is at the center of the circle, it will obtain the same sensor readings regardless of the orientation of the robot. Therefore, as shown in Figure 9(b2), the AAE value at the center of the circle is very high. An open space environment is shown in Figure 9(c1). We can see from its AGM that the farther a position is away from the obstacle, the higher the AAE value. For those cells where the AAE value is higher than one-half of the maximum AAE value (In this AGM experiment for artificial environments, the maximum AAE value is 0.5 m), the AAE value shown in Figure 9(c2) is significantly higher than the AAE value shown in Figure 9(a2,b2). This is because in the open space of Figure 9(c1), a robot actually cannot recognize anything and it cannot localization itself. But in Figure 9(a1,b1), the robot is only confusing in some directions and the sensor reading obtained from those positions can be used to reduce some of the localization uncertainties.

**Figure 10:**
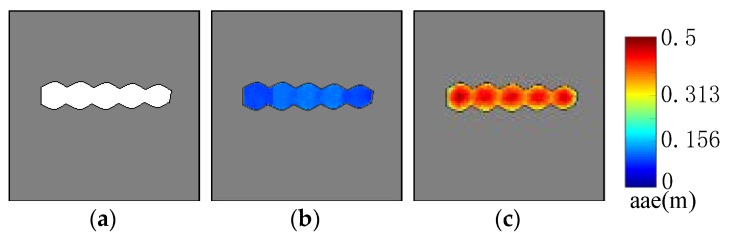
AGMs with different range error for virtual unambiguous environments: (**a**) Occupancy grid map of an unambiguous environment. (**b**) AGM with zero distance error. (**c**) AGM with 0.5 m distance error. The figure clearly shows that large distance error leads to large AAE value.

Figure 10 shows the performance of the AGM of an unambiguous environment under the condition that the simulated laser scan data have different range error. Figure 10a shows the occupancy grid map. Figure 10b,c are the corresponding AGM of Figure 10a when the range error of the laser scan simulator is set to 0 m and 0.5 m, respectively. By comparing Figure 10b with Figure 10c, we can clearly see that even in the same environment, large range error will lead to large AAE value. Although the environment shown in Figure 10a does not seem to be an ambiguous environment, experiment results show that large range error of the laser scan data may still lead to perception aliasing, which demonstrates the importance of sensor noise for localizability evaluation.

### 5.3. AGM of Real Environments

**Figure 11:**
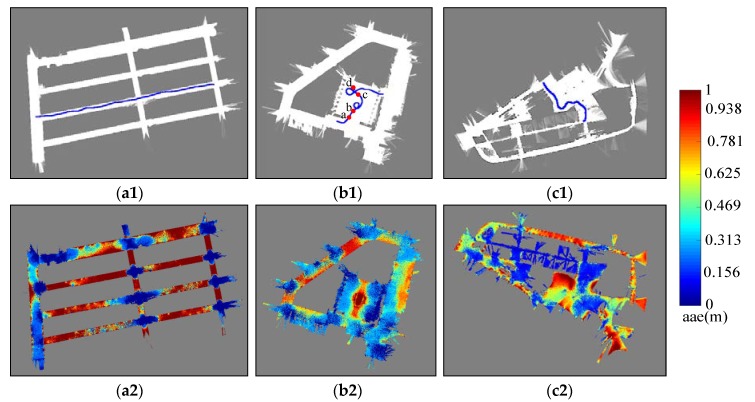
AGMs of real-world environments: (**a1**–**c1**) are the occupancy grid maps of scene A, B and C. (**a2**–**c2**) are the corresponding AGMs. Blue curves show the ground truth for localization simulation. Those positions in the middle of a long corridor, in the center of an open square, near a straight-line wall and tangled thicket get high AAE value.

The SLAM method proposed in [42] was used to build the occupancy grid maps of scene A, B and C which are shown in Figure 11(a1–c1), respectively. In fact, it is difficult to build the occupancy grid maps of these three environments because of their ambiguity. However, with the help of those vehicles parked on the side of the road, localization can be achieved during mapping. Moreover, loop closure and offline optimization adopted by the SLAM method enable us to build consistent occupancy grid maps. After mapping, those vehicles are manually removed from the occupancy grid maps due to the fact that the position of those parked vehicles had been changed when we carried out our localization experiment. The resolution of our occupancy grid maps was set as 0.2 m. In addition, $\chi_{1}$ and $\chi_{2}$ are selected as 0.5 m and 10°, respectively, in our experiments. The true noise parameters of the Hokuyo UTM-30LX 2D Laser range finder are adopted to generate more realistic laser scan data for computing AAE.

It can be seen from the AGM, shown in Figure 11(a2–c2) that those positions which are in the middle of a long corridor, in the center of the open square, near a straight-line wall and tangled thicket get high AAE value. Those positions near a corner get low AAE. The results are in line with human experience.

### 5.4. Localization Simulation

The aim of the simulation experiments is to comprehensively evaluate the performance of our proposed AGM-AMCL method and compare it with the AMCL method and the augmented MCL method proposed in [37]. In fact, the augmented MCL is very similar to the method proposed in [33]. The difference is that extra random particles are added in [37] while sensor resetting method was adopted to generate extra particles in [33], which is not suitable in large scale and ambiguous environments. The trajectories (Figure 11) which are selected to pass through an ambiguous area are used for all simulation experiments. Laser scan data used for localization simulation can be generated at each pose on the simulation trajectories with the help of the occupancy grid maps. $t_{a}$ is set to 0.5 which is the maximum tolerable distance error. $k_{\max}$ is selected to 2 and ρ is set to 0.5. These three parameters were used in all of our experiments. In addition, the localization methods are initialized around the true position in our simulation experiments only to verify the pose tracking performance.

**Figure 12:**
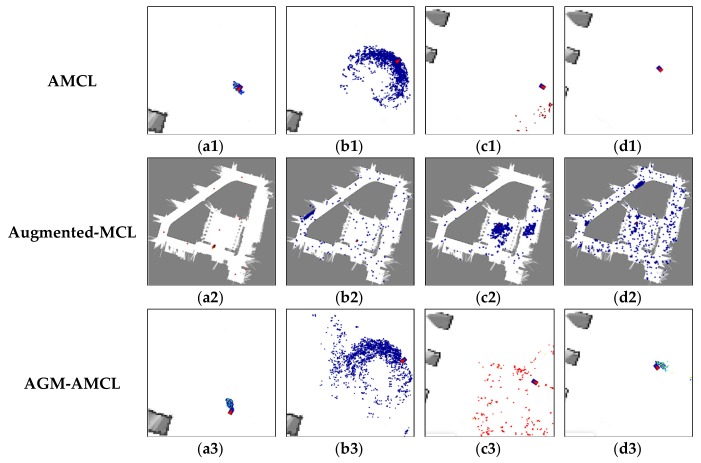
Evolution of particles using AMCL (**top**), Augmented-MCL (**middle**) and AGM-AMCL (**bottom**) in scene B. The letters **a**, **b**, **c** and **d** denote four locations shown in Figure 11(b1) with red dots. The blue square with a red triangle inside represents the true pose and the colored points denote particles. With some particles in the nearby unambiguous area, our method can converge to the true pose after a robot moving through an ambiguous area.

Figure 12 presents the evolution process of particles during localization simulation in scene B. The color of a particle visualizes the particle weight just like the color used in Figure 10 except that the range represented by the color is 0 to 1.

In the beginning, those particles (Figure 12(a1)) generated by AMCL can follow the true pose of the robot but as the robot entering an ambiguous area, the particles (Figure 12(b1)) became divergent and there were fewer and fewer particles (Figure 12(c1)) near the true pose of the robot during the robot moving through an ambiguous area. Even the robot entered an unambiguous area, the particles (Figure 12(d1)) generated by AMCL could not correct the accumulated localization error and converged to a wrong pose. For augmented MCL, extra random particles Figure 12(b2–d2) are added when the average likelihood of particles was decreased. However, those extra random particles led the method to converge to a wrong place in large scale and ambiguous environment. This result shows that it is essential to generate particles in proper locations, otherwise wrong convergence could occur. On the other hand, as the robot entered the ambiguous area, our localization method generate extra particles (Figure 12(c3)) in the adjacent unambiguous area when the accumulated localization error is large enough. Once the robot reached the unambiguous area, those extra particles (Figure 12(d3)) help the robot to correct its accumulated localization error immediately.

**Figure 13:**
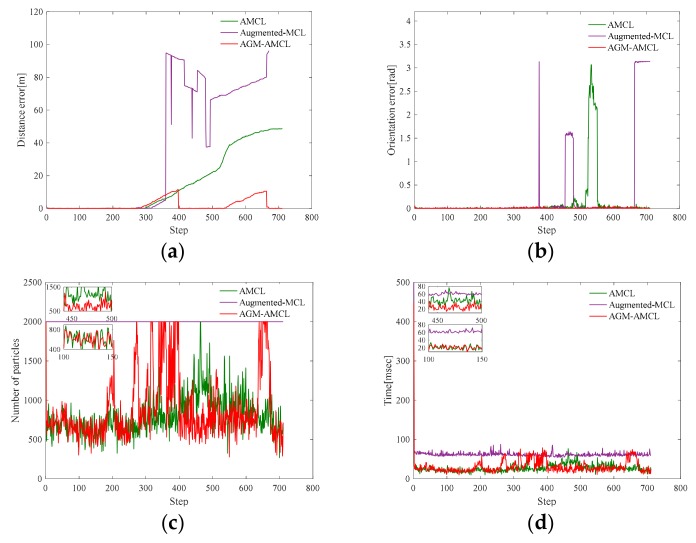
Localization results in scene A: (**a**) distance error; (**b**) orientation error; (**c**) number of particles; (**d**) running time. Note that the orientation errors of AMCL are small in most times. This is because the orientation can be easily determined in the long corridor while it will cause large distance error. The running time of our method is similar to AMCL in unambiguous areas (the zoomed result from step 100 to 150). Our method is more efficient than AMCL after the robot moving through an ambiguous area (the zoomed result from step 450 to 500). Our method needs more particles when the robot is in an ambiguous area and near an unambiguous area (step 300 to 400), therefore it is less efficient than AMCL. Because of the fixed particle number, augmented-MCL takes more time than the other two methods in most time.

**Figure 14:**
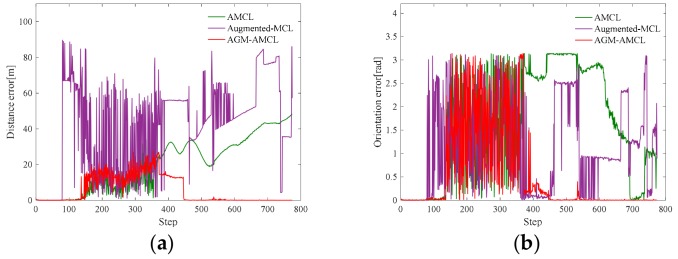

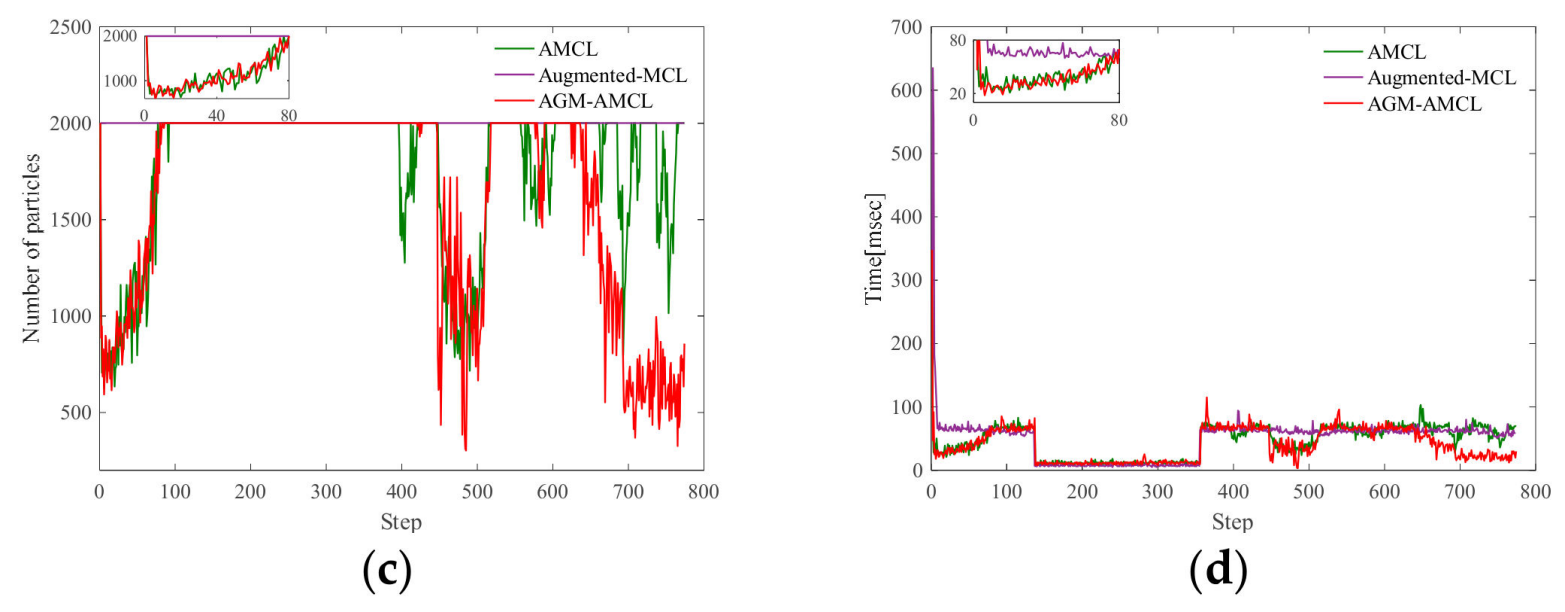
Localization results in scene B: (**a**) distance error; (**b**) orientation error; (**c**) number of particles; (**d**) running time. From step 700 to 800, our method is more efficient because of the right convergence. The zoomed results from step 0 to 80 show that our localization method has similar running time with AMCL in unambiguous area.

**Figure 15:**
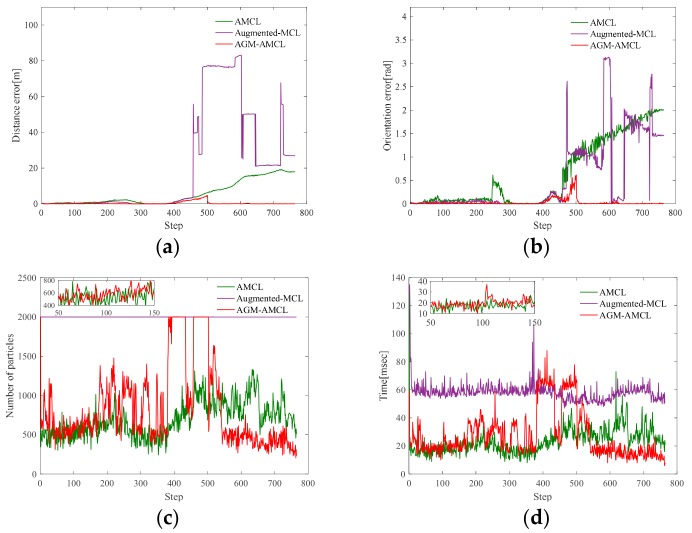
Localization results in scene C: (**a**) distance error; (**b**) orientation error; (**c**) number of particles; (**d**) running time. The zoomed results from step 50 to 150 show that our localization method has similar running time with AMCL. Our method takes less time from step 600 to 800 while more time is need from step 400 to 550.

Figure 13, Figure 14 and Figure 15 present the localization simulation results of three localization methods using the trajectory shown in Figure 11. Distance error and orientation error are shown in (a) and (b). Number of particles and the running time are shown in (c) and (d). In terms of the distance error, our method has better performance than the other two methods. The distance error of AMCL is divergent when the robot moved through the ambiguous areas while the distance error of augmented MCL fluctuate greatly because of the randomly added particles. This is because the orientation can be easily determined in the long corridor while it will cause large distance error. The computational complexity of those three methods is related to the number of particles. However, the particle number of our method and AMCL is stochastic, which rely on the sensor measurement data during robot moving. For this reason, it is hard to evaluate the efficiency theoretically. From the results of three different environments, the efficiency of our method can be demonstrated in three aspects. First, the running time of our method is similar to AMCL in unambiguous environments by using the AGM. Second, because of the right convergence, our method is more efficiency after the robot moving through an ambiguous environment. Third, our method is slower than AMCL when the robot is in an ambiguous area and near an unambiguous area, but it is still enough for real-time localization.

In order to evaluate the localization reliability of our method, we consider the last localization result after a robot traveling through a trajectory. If the distance error of the last localization result is smaller than 0.5 m and the angular error of the last localization result is smaller than 10°, we assume this localization experiment is successful, otherwise, this localization experiment fails. Then the reliability of our localization method can be verified through the localization success rate of repeated localization experiments on the same trajectory. Various degrees of odometer errors were adopted in our reliability experiment. The true odometry data is computed according to:

(22) $$u_{t}^{true}=⊖x_{t-1}^{true}⊕x_{t}^{true}$$

where ⊖ and ⊕ are the pose compounding operator [43], $x_{t-1}^{true}$ and $x_{t}^{true}$ are the true pose of a robot at time t−1 and time t, respectively. $x_{t-1}^{true}$ and $x_{t}^{true}$ can be obtained from the simulated trajectory. Taking the true odometer data as the mean and setting a covariance matrix, a Gaussian distribution can be obtained. The odometer data with errors can be obtained by sampling from such Gaussian distribution. In our experiments, the abovementioned covariance matrix is set to a diagonal matrix with one parameter:

(23) $$\Sigma_{sample}=\left[\begin{array}{ccc} m & 0 & 0\\ 0 & m & 0\\ 0 & 0 & 0.2*m \end{array}\right]$$

Then we define the odometry error rate η  as:

(24) $$\varsigma =\frac{m}{\mathrm{distance}(x_{t}^{true},x_{t-1}^{true})}$$

**Figure 16:**
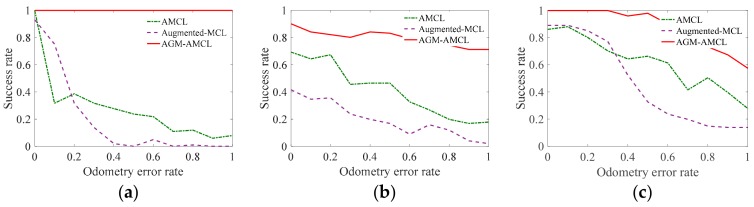
The success rate of three localization methods with different odometry errors: (**a**) Results in Scene A. (**b**) Results in Scene B. (**c**) Results in Scene C. The results show that our method has a considerable success rate even in the case when the odometry error is high in ambiguous environments.

By changing ς, the odometer data with different degrees of error can be obtained. In our reliability experiments, eleven different value of ς varying from 0 to 1 were selected to evaluate the localization reliability under different degree of odometry error. In addition, given ς and a trajectory, we carried out one hundred repetitive experiments to get the localization success rate. The results of the reliability experiments with different odometry errors are shown in Figure 16.

As shown in these results, the localization success rate of AGM-AMCL is significantly higher than that of AMCL and augmented-MCL under different odometry error in three scenes. The minimum success rate of AMCL, augmented-MCL and AGM-AMCL are 6%, 0% and 79%, respectively. Moreover, the localization success rate of AMCL and augmented-MCL decrease rapidly with the increase of odometry error in three scenes while the localization success rate of AGM-AMCL is always 100% in scene A and slightly decreases in scene B and C. The results of the reliability experiments show that our localization method is more reliable when locating a robot in ambiguous environments.

### 5.5. Localization Using Real-World Data

**Figure 17:**
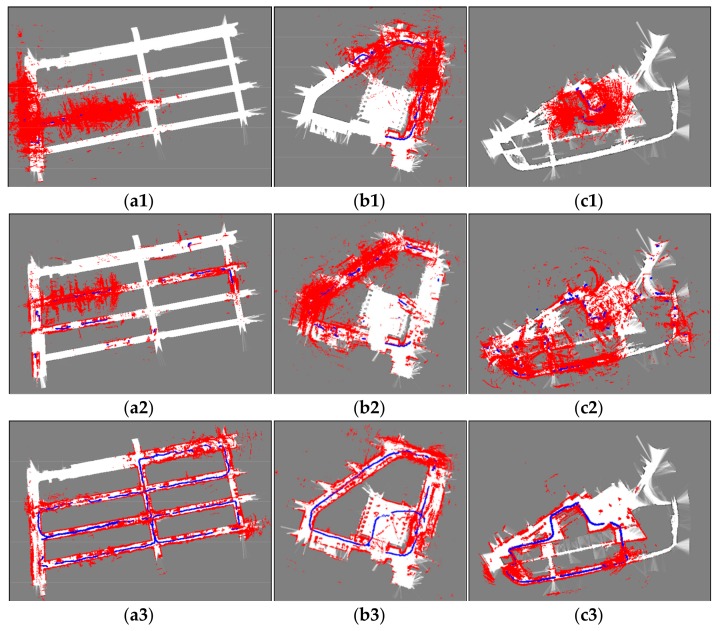
Localization results using real-world data. (**a1**–**c1**) show the localization result using AMCL. (**a2**–**c2**) show the localization result using augmented-MCL. (**a3**–**c3**) show the localization result using our method. Blue curves show the robot trajectories obtained by localization methods. Red point clouds represent the registered laser scan data using the trajectories. Based on the trajectories obtained by our method, laser scan data can be successfully registered in three ambiguous environments. The discontinuity of the trajectories obtained by our method is caused by the portal motion model.

Based on the real-world laser scan data and the odometry data captured by our robot in scene A, B and C, the localization results of three localization methods are obtained, as shown in Figure 17. As we can see from these figures, AMCL and augmented-MCL failed to track the robot pose due to the fact that the registered laser scan data obtained by AMCL and augmented-MCL are inconsistent with the maps. On the contrary, the registered laser scan data obtained by our method is consistent with the maps and the resulting trajectories are closed successfully which are in line with the actual robot trajectories. The inconsistent laser scan data registration obtained by AGM-AMCL is caused by dynamic obstacles and localization error. From the trajectories generated by AGM-AMCL, it can be seen that some discontinuous points appeared on the neighboring locations of an ambiguous area and an unambiguous area. This is because the accumulated localization error was corrected by our localization method after the robot moving through an ambiguous area, which demonstrates that our localization method is more reliable in real-world ambiguous environments.

## 6. Conclusions

In this paper, an AGM-based AMCL method to achieve fast and reliable localization of a robot in ambiguous environments was presented. Our method was derived from an improved DBN which introduces two nodes to represent the accumulated ambiguity error and the AGM separately. The AGM models the ambiguity of an environment, and it can be used to predict the accumulated localization error during pose reasoning. Moreover, utilizing the AGM, a portal motion model was implemented to obtain more reliable pose prediction in an ambiguous area. We evaluated the effectiveness of the AGM and the AGM-AMCL method through simulation and real-world experiments. The results demonstrated that the proposed AGM is in line with human experience and our localization method can achieve fast and reliable localization in ambiguous environments, as compared with conventional methods.

In the future, we will improve the AGM to model the ambiguity in dynamic environments so as to address the localization problem in an environment where the ambiguity is not only caused by symmetrical or featureless areas but also by dynamic objects.
